# A method of radar echo extrapolation based on dilated convolution and attention convolution

**DOI:** 10.1038/s41598-022-13969-6

**Published:** 2022-06-22

**Authors:** Xiajiong Shen, Kunying Meng, Lei Zhang, Xianyu Zuo

**Affiliations:** 1 School of Computer and Information Engineering, Henan University, Kaifeng, Henan China; 2 Henan Provincial Key Laboratory of Big Data Analysis and Processing, Henan University, Kaifeng, Henan China

**Keywords:** Climate sciences, Engineering

## Abstract

The neural network method can obtain a higher precision of radar echo extrapolation than the traditional method. However, its application in radar echo extrapolation is still in the initial stage of exploration, and there is still much room for improvement in the extrapolation accuracy. To improve the utilization of radar echo information and extrapolation accuracy, this paper proposes a radar echo extrapolation model (ADC_Net) based on dilated convolution and attention convolution. In this model, dilated convolution, instead of the pooling operation, is used to downsample the feature matrix obtained after the standard convolution operation. In doing so, the internal data structure of the feature matrix is retained, and the spatial features of radar echo data from different scales are extracted as well. Besides, the attention convolution module is integrated in the ADC_Net model to improve its sensitivity to the target features in the feature matrix and suppress the interference information. The proposed model is tested in the extrapolation of radar echo images in the next 90 min from five aspects—extrapolated image, POD index, CSI index, FAR index, and HSS index. The experimental results show that the model can effectively improve the accuracy of radar echo extrapolation.

## Introduction

Nowcasting was first proposed by Browning^1^ in 1982, and it is mainly used in early warning of disasters like thunderstorms, severe convective weather, rainstorm, and snowstorm. Accurate short-term nowcasting has attracted much attention in the field of meteorological services. Its goal is to accurately and timely predict the local weather in the next 0 to 2 h, enabling weather stations to issue Urban Emergency Disaster Alerts in time. There are three main methods of nowcasting—radar echo extrapolation method, weather situation analysis and forecast method, and comprehensive analysis method, which is the most accurate and widely used method. However, the accuracy of comprehensive analysis method is based on the accuracy of the radar echo extrapolation method. Therefore, the research on the radar echo extrapolation plays a significant role in the forecast and prevention of severe weather.

The traditional radar echo extrapolation methods mainly include tracking radar echoes by correlation (TREC)^2^. The Strom cell identification and tracking (SCIT)^3^ and optical flow^4^. TREC divides two consecutive radar echo images at adjacent times into multiple image subsets, calculates the maximum correlation coefficient of different subsets in the two images, and uses the maximum correlation coefficient to determine the optimal matching area, which is the moving position of the image subset. The TREC method is applicable to extrapolation of steadily changing radar echoes, but not for rapidly changing ones, such as radar echoes of convective precipitation clouds^5^. Based on TREC, Zhang Yaping et al.^6^ proposed difference image based tracking radar echo by correlations (DITREC), which tracks radar echo motion on the basis of the correlation method of differential images. DITREC can eliminate the disordered vector in the TREC vector field due to the rapid change of echoes, but when using the derived DITREC field to predict the precipitation field, the prediction accuracy depends on the Z-I relationship it adopts^7,8^. However, the strong dependence of the Z-I relationship method on a specific climatic background results in the model’s weak generalization ability. Another radar echo extrapolation method, SCIT, calculates the centroid position and volume of the echo monomer, matches the calculated information with the scanned data before and after the echo monomer and track it, and then extrapolates with multiple consecutive moments of the matching and tracking results^9,10^. However, SCIT is only applicable to the identification, tracking and nowcasting of thunderstorm monomers^11^, which limits its application in weather forecasting to a certain extent. The third traditional radar echo extrapolation method, optical flow, finds the corresponding relationship between previous images and the current image by using the change of the target point in the image sequence in the time domain and the correlation between adjacent images. Then the motion information of objects between adjacent images is calculated according to their correspondence. Optical flow obtains the motion vector field of the echo by calculating the optical flow field of the radar echo, and extrapolates the radar echo based on the motion vector field. Different from TREC, the extrapolation of optical flow is based on the changes of radar echoes, rather than the invariant characteristics of the echoes^12^. As a result, optical flow can better track radar echo movement. The extrapolation ability of optical flow is superior to other extrapolation algorithms, especially in rapidly changing weather conditions. However, natural movement is dynamic, so radar echoes will be split, disappear, merge, etc., which is contradictory to the invariance assumption of the generation and disappearance of radar echoes by optical flow, greatly weakening the extrapolation of optical flow. In general, the traditional radar extrapolation method could only infer the echo position of the next moment according to the extrapolated images of several past radar echoes. It ignores the motion nonlinearity of the small and medium-scale atmospheric systems existing in the radar echoes under actual conditions. Therefore, historical radar echo data have not been used to full advantage.

With the development of neural network, its powerful modeling ability and unique advantages in big data processing have attracted extensive attention of scholars, and it has been widely used in the field of meteorology^13^. The application of neural network in meteorology sheds new light on the radar echo extrapolation method, and some scholars have conducted research in this area. Chen Jiahui et al.^14^ used BP neural network model to extrapolate radar echoes and demonstrated the effectiveness of neural network in radar echo extrapolation. Guo Hanyang et al.^15^ used convolutional gated recurrent unit (CGRU) to extrapolate radar echoes and compared them with TREC. The results show that the neural network model has more advantages than traditional methods. Klein^16^ added a dynamic convolutional layer to the convolutional neural networks (CNNs) structure and achieved accurate prediction of precipitation echoes by generating two prediction probability vectors. Based on Klein’s model^17^, added recurrent neural network (RNN) to the dynamic convolutional layer and successfully built recurrent dynamic convolutional neural networks (RDCNN). The neural network has achieved good results in both forecast accuracy and forecast timeliness. As traditional long short-term memory (LSTM) cannot extract spatial features, Shi et al.^18^ replaced the change of LSTM input to state and the change of state to state with convolution in their model and proposed ConvLSTM. To adapt to the spatial–temporal variableness, Shi et al. further improved ConvLSTM in 2017 and proposed TrajGRU^19^. To adapt to the spatio-temporal dependence of radar echo images, Singh et al.^20^ added a convolution structure to recurrent neural network and realized the radar-echo-image-sequence-based prediction. Zhang Lei et al.^21^ proposed an EDP model for sustainable e-agriculture. This weather radar echo prediction method is based on convolution neural network and long short-term memory networks. The results show that the neural network can effectively improve the prediction accuracy and effectiveness. He Liang et al.^22^ regarded precipitation forecasting as a picture-to-picture conversion problem, and used a U-net-structured convolutional neural network for forecasting. To extrapolate radar echoes, Zhang Dezheng et al.^23^ improved the model’s ability to represent the details of spatial structure information by deepening the convolutional layers in the neural network, which is composed of CGRUs.

To sum up, although the application of neural network in radar echo extrapolation is of great significance in utilizing historical radar echo data and improving the accuracy of radar echo extrapolation, the prediction results are still not satisfactory. They could be blurred or distorted, so the prediction accuracy needs to be improved. Therefore, this paper proposes a radar echo prediction model (ADC_Net) based on dilated convolution and attention convolution. To construct small object information and avoid the losing of internal data structure, the model substitutes dilated convolution for pooling in downsampling the feature matrix obtained after convolution. Also, the integration of an attention convolution module with an attention mechanism can improve the model’s sensitivity to target features and suppress interference information, thus enhancing the extrapolation accuracy of radar echoes.

## The model

**Figure 1:**
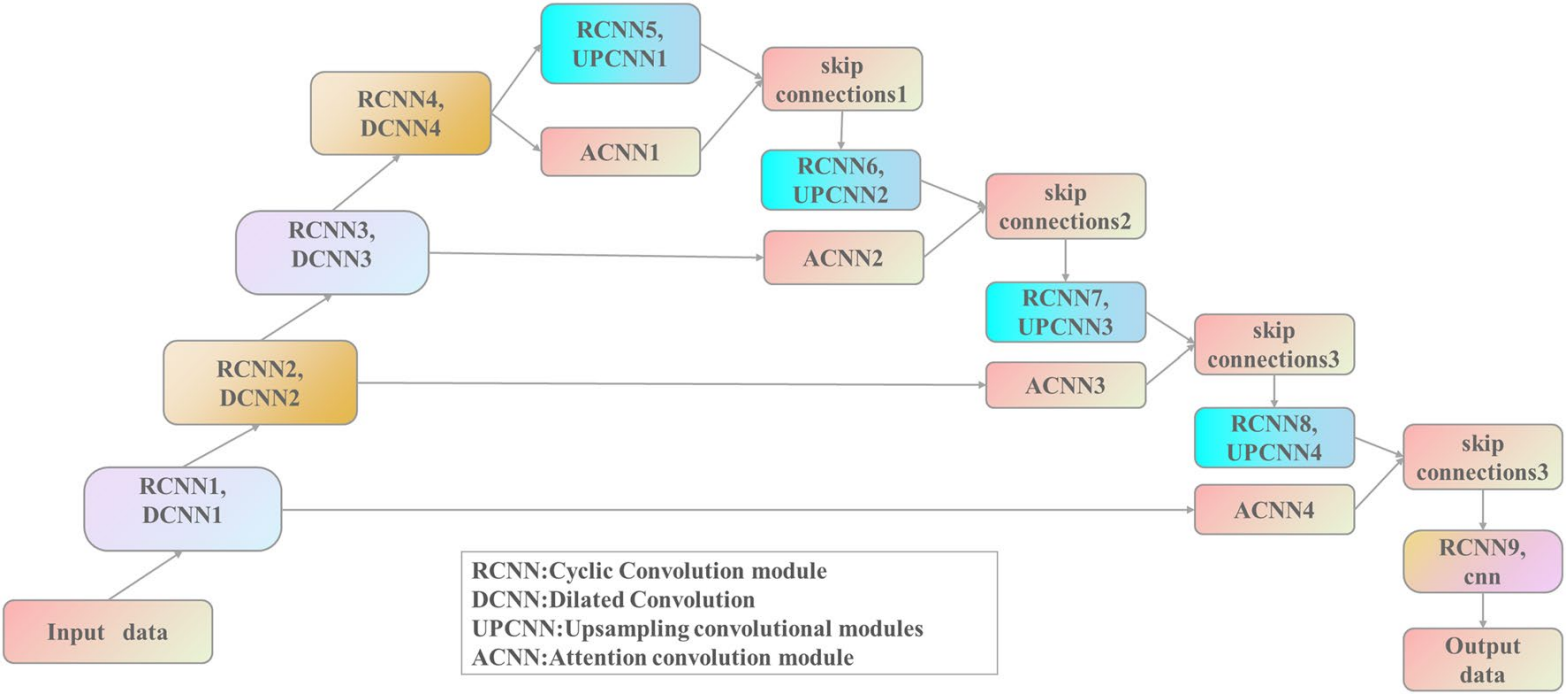
ADC_Net model structure.

The structure of the ADC_Net model based on dilated convolution and attention convolution proposed in this paper is shown in Fig. 1. It follows the encoder-decoder structure, and the network structure is completely symmetric. Firstly, the input image gets a hidden vector through convolution operation, activation function and downsample in the encoder. Then, the hidden vector is progressively upscaled to a higher spatial resolution by an upsampling operation in the decoder. The output is generated when the resolution of the hidden vector equals that of the input image.

**Figure 2:**
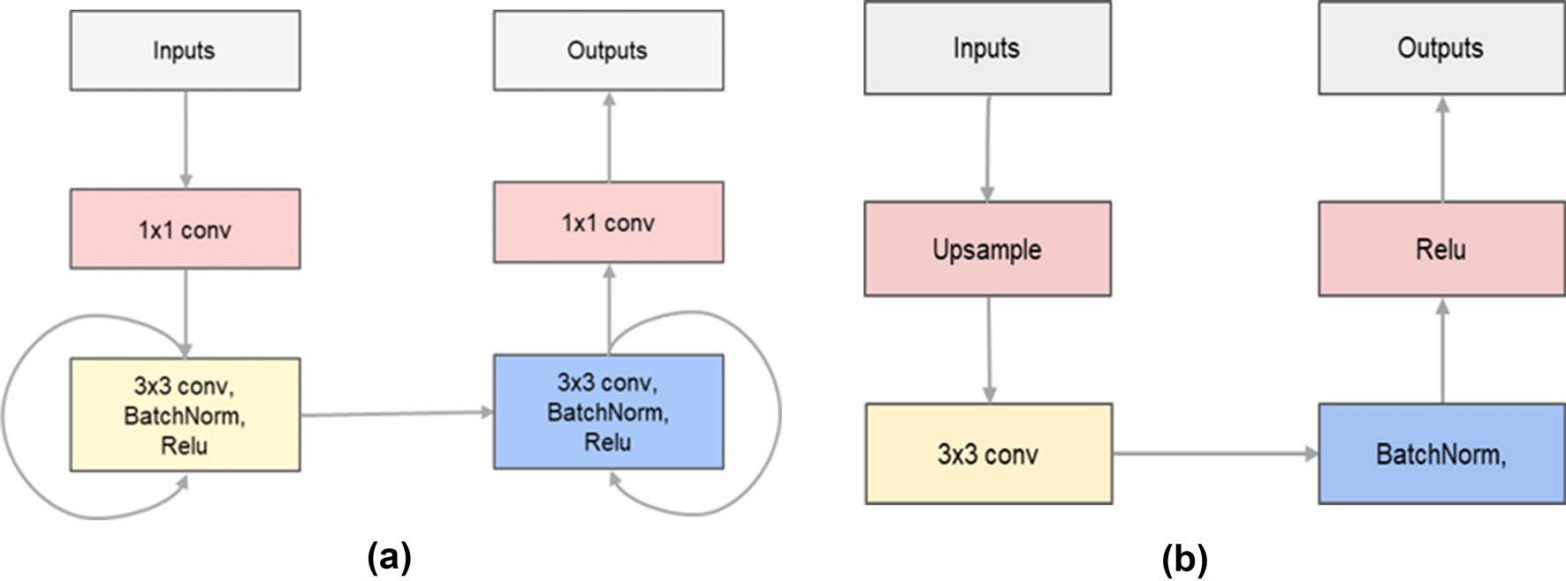
(**a**) RCNN module, (**b**) UPCNN module.

As elementary building blocks, ADC_Net has nine recurrent convolution modules (RCNN), four dilated convolution (DCNN), four upsampling convolution modules (UPCNN), four attention convolution modules (ACNN), four skip connections, and a 1 * 1 standard convolution layer (cnn). RCNN is the basic module of the model, and its structure is shown in Fig. 2a. Because the key to radar echo extrapolation is the prediction of echo spatio-temporal sequence, RCNN has a recurrent memory unit that solves the temporal memory problem and a convolutional unit that extracts spatial features. Therefore, using the RCNN module in the model can enhance its ability to capture the changing laws of radar echoes. Dilated Convolution (DCNN) is used to downsample the feature matrix instead of pooling operation. In the training process, the model can not only extract the spatial features of radar echo data from different scales, but also retain the internal data structure of the feature matrix after downsample. It avoids the problems of the pooling operation, such as losing internal data structure and being unable to reconstruct information of small objects. The upsampling convolution module (UPCNN) is used to gradually restore the hidden vector obtained in the neural network encoder to the same size as the input data. The specific module structure is shown in Fig. 2b. The attention convolution module (ACNN) assigns different weights to each pixel of the feature matrix during model training and prediction. The pixel information with stronger correlation with the current radar echo extrapolation task is given a higher weight value, and the pixel information with weaker correlation with the current radar echo extrapolation task is given a lower weight value. The skip connections combine the weight matrix obtained by ACNN with the high-level feature matrix in the decoder, and assign the attention weight to the high-level feature matrix, thereby improving the model's sensitivity to pixel information related to the current radar echo extrapolation task and suppressing irrelevant pixel information.

In the above module, dilated convolution instead of pooling operation is employed to downsample the feature matrix in the model and the attention convolution module is used to improve the model's sensitivity to the target features in the feature matrix and suppress the interference information. As the dilated convolution and the attention convolution module are the two main innovative structures of the ADC_Net model proposed in this paper, they will be illustrated in the following paragraphs.

### Dilated convolution

**Figure 3:**
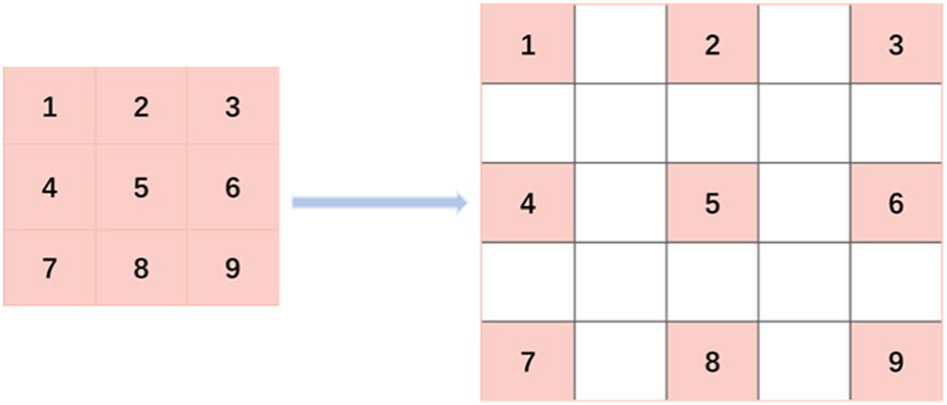
Difference between convolution kernels of dilated convolution and standard convolution.

Dilated convolution, also known as convolution kernel expansion operation, is a convolution method commonly used in pixel-wise output models^24^. To increase the reception field, dilated convolution injects holes on the basis of the convolution map of standard convolution. Therefore, dilation rate, a hyper-parameter, is added to dilated convolution. The hyper-parameter refers to the number of intervals in the kernel, meaning filling the dilation rate-1 zeros in the convolution kernel. Figure 3 shows the difference between the convolution kernels of dilated convolution and standard convolution. The left side represents the 3 * 3 convolution kernel of standard convolution, and the right side represents the 3 * 3 convolution kernel of dilated convolution with the dilation rate of 2. The middle white part of the convolution kernel is filled with 0, and the unilateral size of the convolution kernel of dilated convolution becomes 2 * (3 − 1) + 1 after filling, as is shown in formula (2).

**Figure 4:**
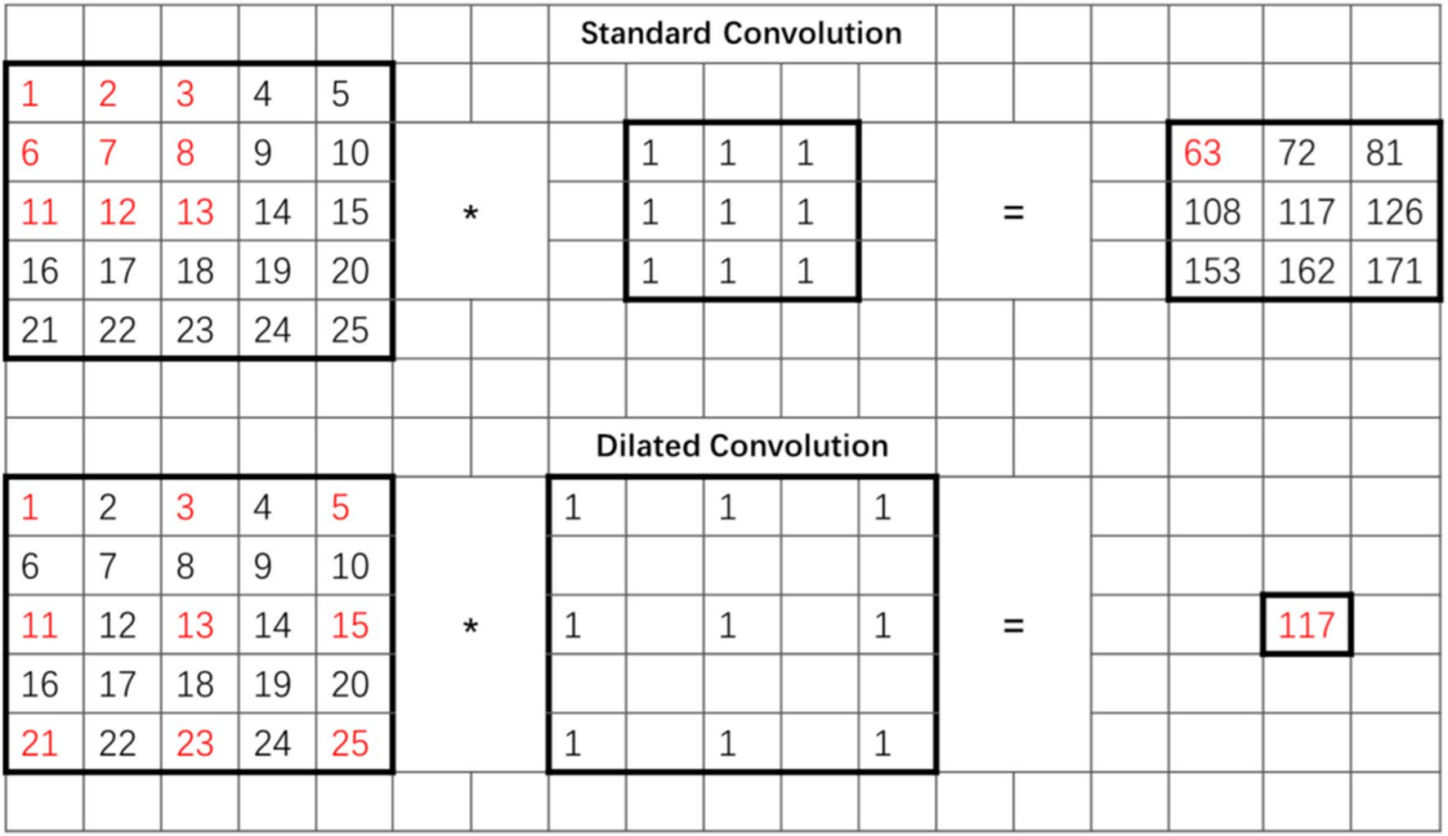
The difference between the reception field and the output of standard convolution and dilated convolution.

Dilated convolution expands the convolution kernel to the scale of the dilated scale constraint, and fills the area not occupied by the original convolution kernel with zeros. This ensures the exponential expansion of the reception field by dilated convolution without losing resolution or coverage. Therefore, in dilated convolution, the size of the feature matrix before and after convolution is not only related to the convolution kernel, convolution step size, and padding, but also affected by the dilation rate. For standard convolution, the calculation method of the feature matrix size after the convolution layer is shown in Eq. (1). Here, h1 represents the length (or width) of the input feature map; K is the size of the convolution kernel; p is the padding value; s represents the stride; and h2 represents the length (or width) of the output feature map. When calculating the size of the feature map of dilated convolution after passing through the convolutional layer, the influence of the dilation rate on the size of the convolution kernel should be considered first. Equation (2) is the calculation formula of the size of the convolution kernel after expansion; ke is the size of the convolution kernel after the expansion; k is the size of the convolution kernel before the expansion; and r is the expansion coefficient. Equation (3), which combines (1) and (2), is the size calculation formula of the feature map after the convolution operation of dilated convolution. Figure 4^25^ takes a 3 * 3 convolution kernel as an example to show the difference between standard convolution and dilated convolution in the reception field and output. The dilation rate of dilated convolution is 2; the red part of the two figures on the left is the eigenvalue of the convolution operation with the convolution kernel, and the red part on the right is the output result after convolution.

1 $${\text{h2 }} = ({\text{h}}1 - {\text{k}} + {\text{2p}})/{\text{s }} + { 1}$$

2 $${\text{ke }} = {\text{ r }}({\text{k}} - {1}) + {1}$$

3 $${\text{h2 }} = ({\text{h1}} - {\text{ke}} + {\text{2p}})/{\text{s }} + { 1} = [{\text{h1}} - {\text{ r }}({\text{k}} - {1}) - {1} + {\text{2p}}]/{\text{s}} + {1}$$

When using a neural network to process images, features are generally extracted through convolution operations in the neural network, and then the convolutional feature matrix is downsampled by a pooling operation to reduce the image scale and increase the reception field. However, the loss of information caused by downsampling in the pooling operation is irreversible, and this irreversible loss of information will cause the loss of the internal data structure of the feature matrix during the training process of the neural network. Usually only the probability of each class needs to be predicted in the classification model, so there is no need to consider the loss of image details caused by the pooling operation. When doing the pixel-level prediction task of radar echo extrapolation, it is necessary to restore the image to the same size as the input image through the upsampling operation, so the problem of irreversible information loss needs to be reconsidered. Therefore, in order t to keep resolution and still expand the receptive field, in this paper, dilated convolution, instead of the pooling operation, is used to downsample the feature matrix obtained after convolution in the model, and the reception field of the convolution kernel is expanded through dilated convolution without losing resolution or coverage. In this way, dilated convolution, together with standard convolution, can not only perform convolution operation on data and extract spatial features from radar echo data from multiple scales, but also retain internal data structure, making data information reconstruction possible, which cannot be realized by pooling operation. This is useful in radar echo extrapolation tasks. On the one hand, a larger reception field makes it possible to better learn the overall change trend of radar echoes, and on the other hand, a higher resolution makes it possible to accurately locate the pixel information of smaller radar echoes.

### Attention convolution module

**Figure 5:**
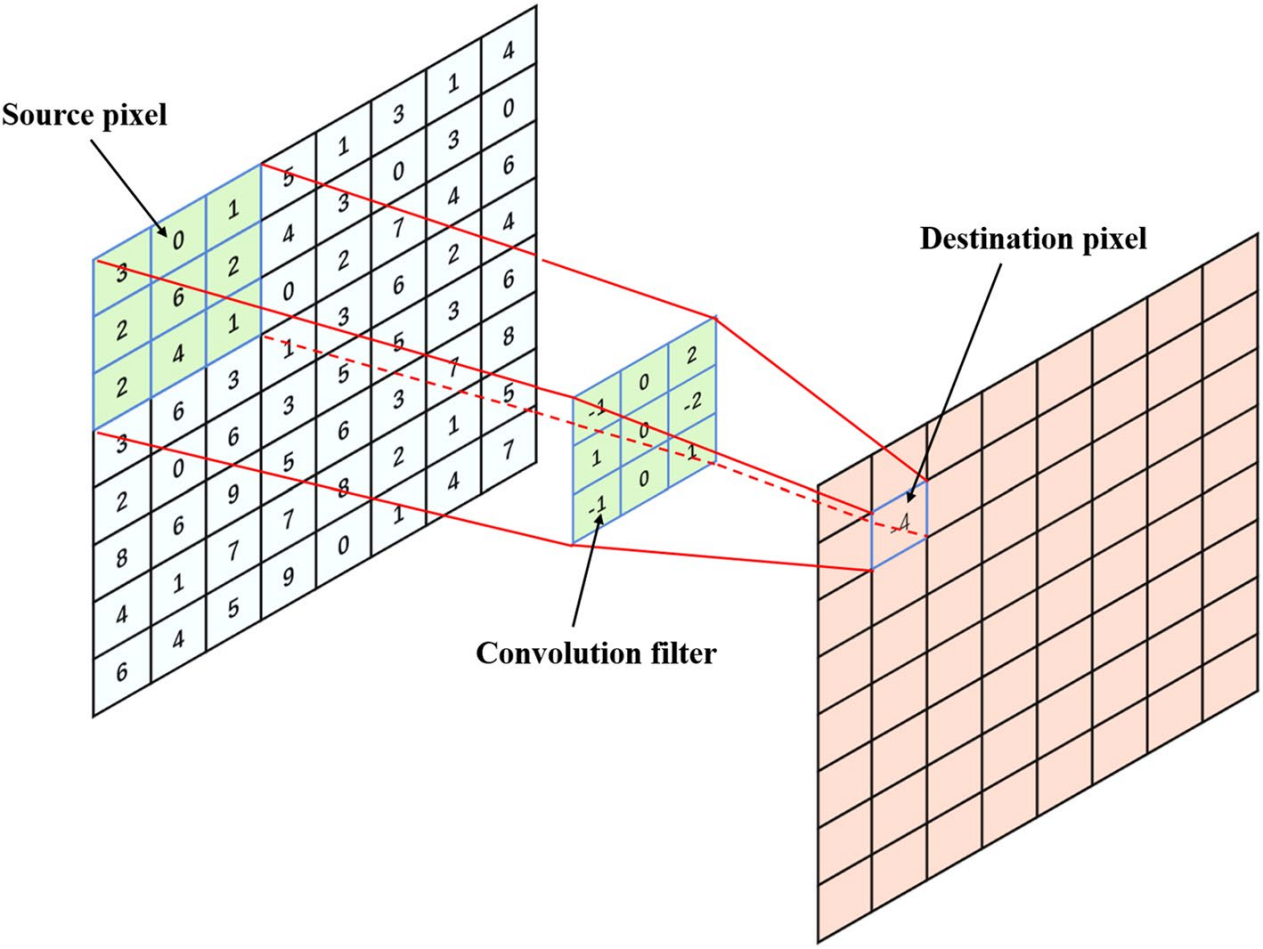
Example of information limitations of convolution operations.

The attention mechanism has gradually become a research hotspot and an important part of the neural network structure in recent years. Initially applied to machine translation, now it has been used in image processing and recommendation systems^26,27^. The attention mechanism comes from the human visual attention mechanism. Humans usually observe and pay attention to a specific part of a scene according to the needs instead of seeing a scene throughout. Therefore, the essence of the attention mechanism is to filter out important information from a large amount and focus on them. In the neural network, a hidden vector is generated by the input image through convolution operation, activation function and pooling operation. The value of this hidden vector is calculated by referring to itself and several surrounding pixels. As a result, if standard convolution is employed, local information rather than global information will be used to calculate target pixels, which brings limitations to the training of neural networks, as is shown in Fig. 5. In the 3 × 3 convolution operation (Fig. 5), the convolution filter has 9 pixels, and the value of the target pixel is calculated only with reference to itself and the surrounding 8 pixels, while the global pixel information does not play a role, which causes deviation in prediction results. Consequently, with increasing neural network layers, the high-level feature map has strong predictive ability, but the target is blurred^28^. In contrast, low-level feature maps have more accurate target location but poor prediction ability and more virtual target information^29^.

**Figure 6:**
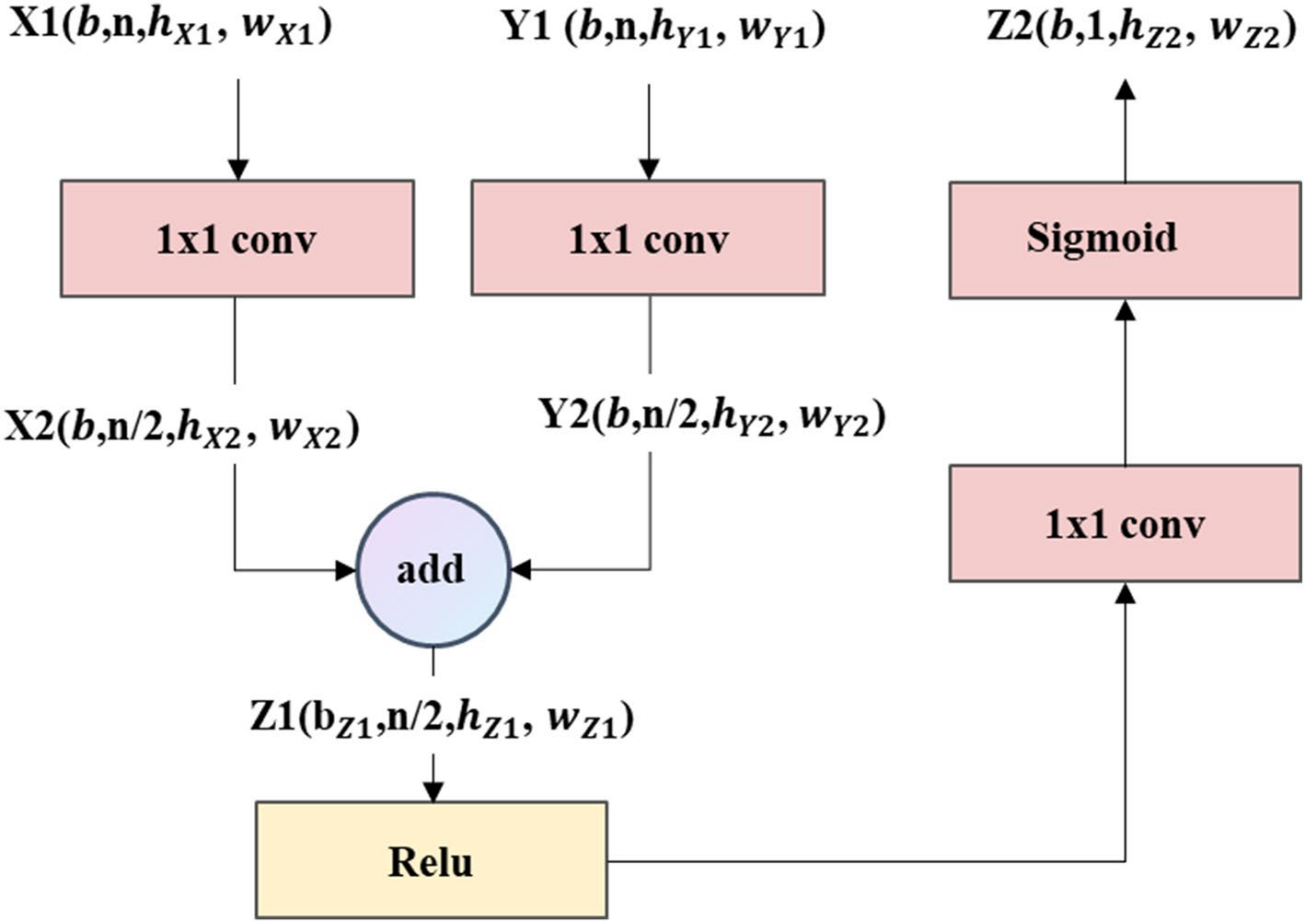
Attention Convolution module structure.

In order to solve the above problems, this paper integrates the advantages of high-level feature maps and low-level feature maps. The attention convolution module is added to the ADC_Net neural network model. Considering each pixel in the feature map as a random variable, calculate the weight values of all pixels in the current prediction task, the pixels with high weights are used and the pixels with low weights are ignored in training and prediction. The use of the attention convolution module can effectively suppress the pixel information irrelevant to the current prediction task and enhance the related pixel information. Figure 6 shows the structure of the attention module used in this paper, where b is the batch size of the model, i.e., the number of samples selected for one training, h and w the height and width of the feature matrix, and cat means matrix addition. First, two feature matrices with n channels, high-level feature matrix X1(b,n,${h}_{X1}$,${w}_{X1}$) and low-level feature matrix Y1(b,n,${h}_{Y1}$, ${w}_{Y1}$) are input into the attention convolution module. In the attention convolution module, the feature information of different channels is integrated through 1 × 1 convolution, and the number of channels of the feature matrix is halved to reduce the amount of model computation. At this point, feature matrices X2(b,n/2,${h}_{X2}$,${w}_{X2}$) and Y2(b,n/2,${h}_{Y2}$,${w}_{Y2}$) are obtained. Then calculate the sum of the two characteristic matrices of X2(b,n/2,${h}_{X2}$,${w}_{X2}$) and Y2(b,n/2,${h}_{Y2}$,${w}_{Y2}$) to get the characteristic matrix Z1(${\text{b}}$,n/2,${h}_{Z1}$, ${w}_{Z1}$). The nonlinearity of the neural network is then increased through the Relu activation function. Finally, Z1 undergoes a 1 × 1 convolution and a sigmoid activation function to obtain a weight matrix Z2 (${\text{b}}$,1,${h}_{Z2}$, ${w}_{Z2}$) with channel number of 1 and value range of (0, 1) and output. The specific calculation process of the weight of each pixel is shown in Eqs. (4) and (5). ${W}_{X1}, {W}_{Y2}, {W}_{Z1}$ are the convolution kernels of the feature matrix X1, X2, Z1. xϵX2(b,n/2,${h}_{X2}$,${w}_{X2}$),yϵY2(b,n/2,${h}_{Y2}$, ${w}_{Y2}$), Z1 $\in Z1(\mathrm{b},\mathrm{n}/2,{h}_{Z1}, {w}_{Z1})$, Z2 $\in Z1(\mathrm{b},\mathrm{n}/2,{h}_{Z2}, {w}_{Z2})$. u, v, i, j are the indices of pixel x and pixel y in X2 and Y2, respectively, where uϵ(0,b), vϵ(0,n), iϵ(0,h), j ϵ(0,w). The final weight of each pixel, ${Z2}_{i,j}^{u,v}$ is calculated by Eqs. (4) and (5). ${Z2}_{i,j}^{u,v}$ is used for each pixel to determine focus regions. The smaller the value of ${Z2}_{i,j}^{u,v}$ is, the smaller the pixel weight value of the position is. When the weight value of a certain pixel is close to 0, it is considered that the pixel has nothing to do with the current prediction task and is interference information. On the contrary, the larger the obtained value of ${Z2}_{i,j}^{u,v}$, the larger the weight value of the pixel at the position, and the position of the pixel is considered to be the focus region.

4 $${Z1}_{i,j}^{u,v}=max \left(0,\left({W}_{X1}{x}_{i,j}^{u,v}+{W}_{Y1}{Y}_{i,j}^{u,v}\right) \right)$$

5 $${Z2}_{i,j}^{u,v}= \frac{1}{1+exp \left(-\left({W}_{Z1}{Z1}_{i,j}^{u,v}\right) \right)}$$

To sum up, the application of the attention convolution module can simultaneously learn the advantages of high-level feature maps and low-level feature maps in the process of model training and prediction to achieve the global reference of feature values. According to the weight matrix obtained by referring to the global feature, each pixel of the feature matrix is given different weights. This allows the neural network to focus on the pixel information most relevant to the current task each time, making the neural network use pixel information more efficiently in the process of training and prediction^21^, so the prediction results are more accurate.

## Experiments

### Data

**Figure 7:**
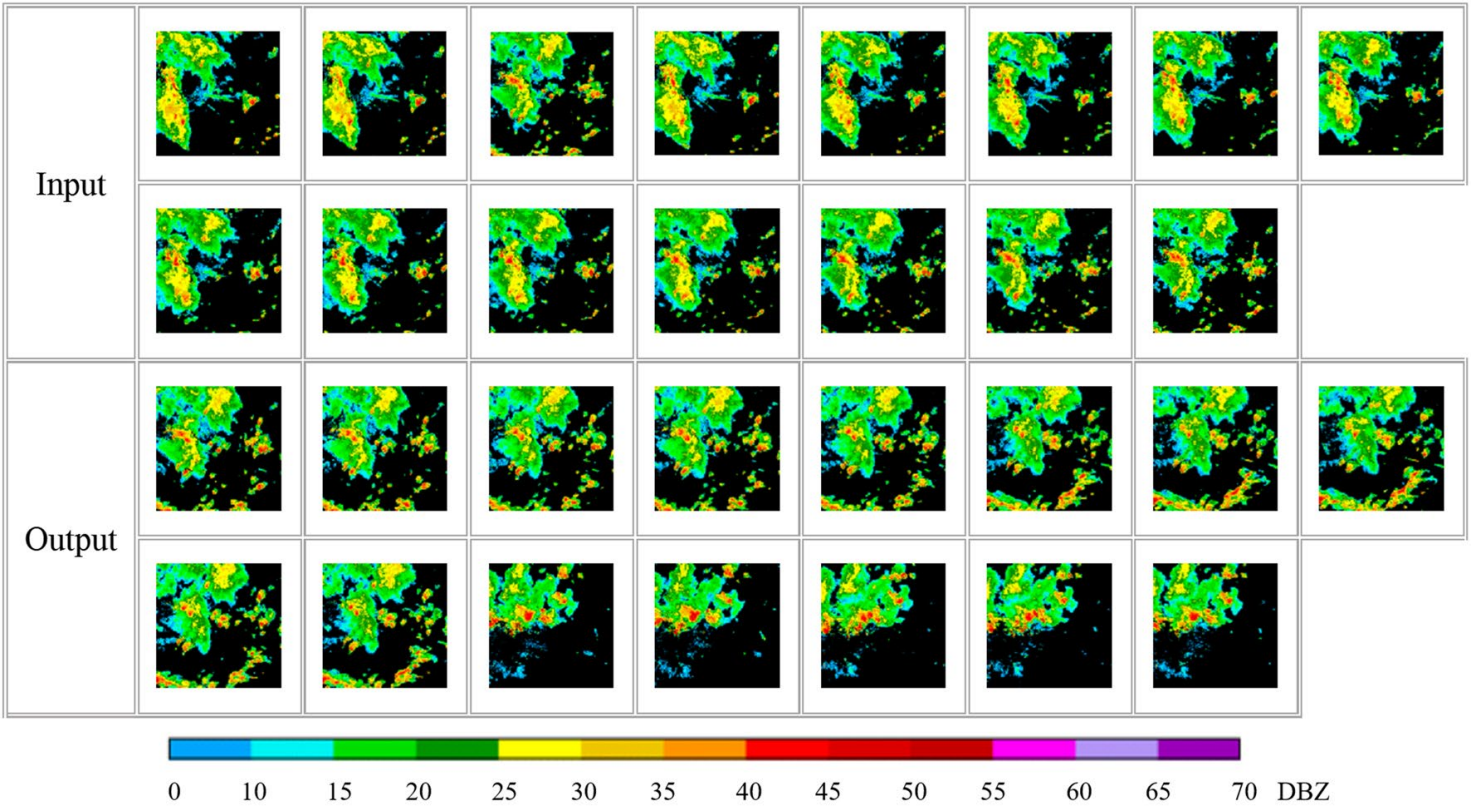
Image after visualization of a set of samples in the dataset.

The data in the experiment are New generation of Doppler weather radar detection data, which are stored in polar coordinates. For the convenience of research, the radar echo data in polar coordinates are first stored in the three-dimensional Cartesian coordinate system by interpolation method. Considering the height of the representative average guided airflow in the troposphere, we select the CAPPI radar echo image at a vertical height of 3KM as the experimental data. In order to reduce the parameters in the training process and improve the training efficiency, the CAPPI images are grayscaled before the experiment. The processed grayscale image is then cropped, keeping a 256 * 256 size area in the center of the image. Because the echoes with areas too small are generally weak and evolve rapidly, they have little guiding significance for forecasting work. In order to avoid the influence of the sample quality on the training results, each radar echo image is screened during the construction of the sample dataset. The cases with the echo area coverage greater than 1/10 in the effective detection range of the radar are selected into the sample data set, and the radar echo range is controlled within the range of 0–70 DBZ. Figure 7 shows the visualized images of a group of samples in the data set. The first two rows in Fig. 7 are fifteen input images, and the last two rows are fifteen label images.The time interval between each two adjacent radar echo images in the data used in this paper is 6 min. In this experiment, the first fifteen radar echo images are used to extrapolate the last fifteen radar echo images. That is, the radar echo image of the first 90 min is used to predict the radar echo image of the next 90 min.

### Model evaluation indicators

In the model training process, the prediction of strong convective weather is used as an example to evaluate the experimental results. The evaluation indicators are probability of detection (POD), false alarm rate (FAR), critical success index (CSI) and Heidke skill score (HSS). POD represents the probability that convective weather is predicted to occur and the prediction is successful. FAR represents the probability that convective weather is predicted to occur but the prediction fails. CSI is the prediction accuracy after weighing successful predications and wrong predications. HSS is a quantitative evaluation of the model after comprehensively considering various situations. Therefore, the higher the value of POD, CSI and HSS is, and the lower the value of FAR is, the more accurate the predicted result is, and the better the model effect is. Since strong convective weather usually occurs when the radar echo intensity is greater than 35DBZ, the threshold of the radar echo is set to 35DBZ in this experiment. The real label radar echo image and the predicted radar echo image are binarized respectively, with the pixels larger than 35DBZ in the radar echo image binarized to 1, and the pixels smaller than 35DBZ binarized to 0. When evaluating the experimental results, the binarized pixel value of the real image is named label, and the binarized pixel value of the predicted image is named fake, *hit* shows the times that both the predicted value fake and the real value label are 1, *far* is the times that the predicted value fake is 1 while the true value label is 0, *miss* the times the predicted value fake is 0 but the true value label is 1, and *cn* is the times that both the predicted value fake and the true value label are 0. The calculation formulas of the experimental evaluation indicators POD, FAR, CSI and HSS are shown in Eqs. (6), (7), (8), and (9), respectively.

6 $$POD= \frac{hit}{hit+miss}$$

7 $$FAR= \frac{far}{hit+far}$$

8 $$CSI= \frac{hit}{hit+miss+far}$$

9 $$HSS= \frac{2*(hit*cn-miss*far)}{\left(hit+miss\right)*\left(miss+cn\right)+\left(hit+far\right)*(far+cn)}$$

### Loss function

The loss function is the most basic and critical structure in deep learning, and is used to evaluate the degree of inconsistency between the predicted value of the model and the true value. It is a non-negative real-valued function, usually represented by L (s, y), where s and y represent the predicted value and the true value, respectively. In the process of neural network training, the smaller the loss function value is, the more robust the model is. The prediction effect of the model can be measured through the definition and optimization of the loss function. In model training, considering that the use of the MSE loss function alone may cause a small loss value and result in a poor model training effect, the use of MAE alone may lead to the loss of data feature details. Therefore, in this paper, MSE + MAE is used as the loss function, which is conducive to the convergence of the function. Even if the learning factor is fixed, the function can quickly obtain the minimum value. The loss function of the proposed model is shown in Eq. (10).

10 $$L\left(S,Y\right)=MSELoss\left(s,y\right)+MAELoss\left(s,y\right)= \frac{1}{n}\sum_{t=1}^{n}{({s}_{t}-{y}_{t})}^{2}+\frac{1}{n}\sum_{t=1}^{n}|{s}_{t}-{y}_{t}|$$

### Optimizer

During the training and testing of the neural network, the choice of different optimizers will impact the prediction results differently. In this paper, Adam is used as the optimizer. Integrating the advantages of the two optimization algorithms AdaGrad and RMSProp, Adam calculates the update step size by considering the First Moment Estimation of the gradient (the mean of the gradient) and the Second Moment Estimation (the uncentered variance of the gradient). Its advantages include easy operation, high computational efficiency, and parameter updates immune to the scaling transformation of gradients. Also, because of the good interpretability of Adam hyperparameters and little or no need for parameter adjustment, it is applicable to large-scale data and parameter scenarios. In this experiment, the initial learning rate of Adam is set to 0.0001.

### Analysis of experimental results

The language used in this experiment is Python, and the Pytorch framework is used for training on NVIDIA 2488 GPU. To evaluate the performance of the ADC_Net model based on dilated convolution and attentional convolution (ADC_Net) proposed in the paper, it is compared with the ADC_Net model based on dilated convolution (ADC_Net2), the ADC_Net model based on attention convolution module (ADC_Net3), the ADC_Net model based neither on dilated convolution nor on attentional convolution module (ADC_Net4), U_Net, EDP model, and ConvLSTM.

#### Evaluation of model prediction results

**Figure 8:**
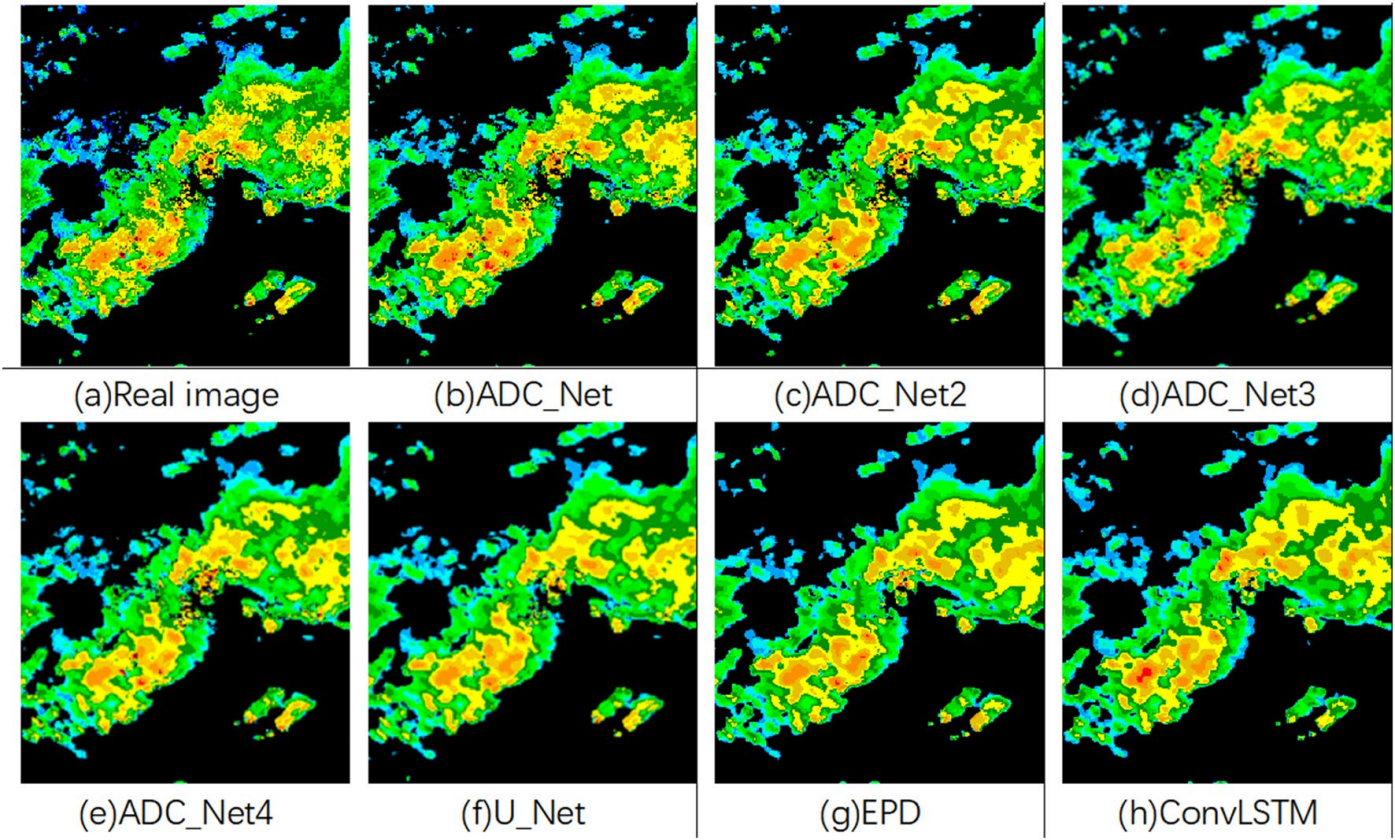
Comparison of model radar echo extrapolation results.

Figure 8 shows the comparison between the real radar echo image and the radar echo image predicted by each model. It can be seen that the predicted image of the ADC_Net model based on attention convolution and dilated convolution proposed in this paper is most similar to the real image. In comparison, although U_Net, EDP model, and ConvLSTM can also predict the shape of the radar echo with accuracy, they could only show a general outline with blurred details in terms of radar echo intensity. Compared with ADC_Net4, the prediction results of ADC_Net3 are more accurate in details, such as small gaps between radar echoes. This shows that adding an attention convolution module allows the model to learn more detailed features of the data. Compared with ADC_Net2, the predication results of ADC_Net3 are not so impressive for the high radar echo intensity areas (shown in red in Fig. 8). It is because dilated convolution instead of pooling is used in ADC_Net2 to downsample and retain the features’ internal data structure, which allows information reconstruction to keep detailed information of the image, while this cannot be realized by pooling.

Tables 1, 2, and 3 show the POD evaluation scores, CSI evaluation scores, FAR evaluation scores, and HSS evaluation scores of radar echo extrapolation at 30 min, 60 min, and 90 min for all models.With higher value of POD, CSI and HSS, and lower value of FAR, the predicted result is more accurate and the model is more effective. It can be clearly seen that the scores of each evaluation index of the ADC_Net model based neither on dilated convolution nor on attentional convolution module (ADC_Net4) are similar to U_Net, EDP model, and ConvLSTM. However, when dilated convolution (ADC_Net2) or attention convolution module (ADC_Net3) is added to the model, the evaluation scores of each indicator are significantly better than that of U_Net, EDP model and ConvLSTM. It can be seen from the experimental evaluation scores of ADC_Net2 and ADC_Net3 that both the dilated convolution and the attention convolution module can improve the accuracy of radar echo extrapolation in the radar echo extrapolation task. When the dilated convolution and attention convolution modules are added to the model (ADC_Net) at the same time, the POD, CSI, HSS evaluation scores of the model in the three time periods and the FAR evaluation score in 30 min are the best. Although the FAR values at 60 min and 90 min are not as good as ADC_Net2 and ADC_Net3, they are also much better than ADC_Net4 and U_Net, EDP, and ConvLSTM. This shows that for the radar echo extrapolation task, the dilated convolution and attention convolution modules can be put in the same model, and the integration of the two can achieve better results than using dilated convolution or attention convolution alone.

**Table 1:** 30-min radar echo extrapolation evaluation scores.

	30 min			
	POD	CSI	FAR	HSS
ADC_Net	0.76087586	0.58172320	0.22049363	0.60302877
ADC_Net2	0.72091937	0.52216042	0.24340544	0.59199967
ADC_Net3	0.70376852	0.53149514	0.23615935	0.55343548
ADC_Net4	0.62247112	0.47155796	0.27031753	0.53146832
U_Net	0.59286521	0.44365503	0.30021739	0.50110344
EDP	0.61163434	0.46923196	0.27989904	0.45632585
ConvLSTM	0.56044080	0.42366371	0.29077875	0.45303934

**Table 2:** 60-min radar echo extrapolation evaluation scores.

	60 min			
	POD	CSI	FAR	HSS
ADC_Net	0.73668836	0.55113560	0.25978267	0.54246716
ADC_Net2	0.68287509	0.49765180	0.25450861	0.54545296
ADC_Net3	0.65736475	0.48938651	0.25422885	0.53725577
ADC_Net4	0.59543897	0.42196212	0.32134102	0.50585725
U_Net	0.52786379	0.40746430	0.31627907	0.45462281
EDP	0.55468666	0.42866114	0.31237780	0.44512617
ConvLSTM	0.52867036	0.41107007	0.3188	0.41275907

**Table 3:** 90-min radar echo extrapolation evaluation scores.

	90 min			
	POD	CSI	FAR	HSS
ADC_Net	0.69663783	0.50318873	0.297786948	0.51354401
ADC_Net2	0.60519550	0.45486333	0.276377305	0.50323715
ADC_Net3	0.61885704	0.42953144	0.307918722	0.50190045
ADC_Net4	0.54688346	0.40419994	0.349850822	0.47244314
U_Net	0.41271537	0.40040904	0.39193696	0.40240053
EDP	0.49411441	0.40056530	0.365196424	0.40518047
ConvLSTM	0.41460854	0.40004941	0.392364732	0.40312401

#### Model parameter performance evaluation

This part compares the single training time and model parameters of ADC_Net model based on the ADC_Net model based on dilated convolution and attention convolution (ADC_Net), the ADC_Net model based on dilated convolution (ADC_Net2), the ADC_Net model based on attention convolution module (ADC_Net3), the ADC_Net model based neither on dilated convolution nor on attentional convolution module (ADC_Net4) and U_Net, EDP model, ConvLSTM. The details are shown in Table 4. As can be seen from Table 4, although the EDP model has the most parameters, the running time is the fastest and the training efficiency is the highest. Compared with U_Net and ConvLSTM, the model proposed in this paper can improve the prediction accuracy of radar echo extrapolation, but it has more model parameters and a longer single prediction time. The one with the most parameters and the slowest running is ADC_Net2, which may be a result of the preservation of the internal data structure when the data is downsampled with dilated convolution. However, by comparing the model parameters and running time of ADC_Net2 and ADC_Net, it can be found that when both dilated convolution and attention convolution are added to the model, the parameters in the model training process are reduced, and the training time is also significantly shortened. This shows that combining attention convolution with dilated convolution can not only improve the prediction performance of the model, but also reduce the parameters and running time added by dilated convolution to the model to a certain extent through the attention convolution module.

**Table 4:** Performance comparison.

	ADC_Net	ADC_Net2	ADC_Net3	ADC_Net4	U_Net	EDP	ConvLSTM
Model parameters (unit: pcs)	39,444,603	40,485,711	38,483,036	39,093,071	34,534,863	79,713,680	29,396,039
Single training time (unit: s)	12.46	13.38	12.25	12.31	8.94	7.34	7.81

## Conclusion

This paper proposes a radar echo extrapolation model (ADC_Net) based on dilated convolution and attention mechanism. Using dilated convolution instead of pooling operation can not only perform spatial feature extraction on radar echo data from multiple scales, but also preserve the internal data structure. It avoids the loss of the internal data structure in pooling and makes reconstruction of small object information possible. Also, by integrating an attention convolution module with attention mechanism into the model, the sensitivity to the target features is improved, and the interference information is suppressed, thereby improving the extrapolation accuracy of radar echoes. The radar echo extrapolation results of the ADC_Net model based on dilated convolution and attentional convolution (ADC_Net) proposed in the paper is compared with that of the ADC_Net model based on dilated convolution (ADC_Net2), the ADC_Net model based on attention convolution module (ADC_Net3), the ADC_Net model based neither on dilated convolution nor on attentional convolution module (ADC_Net4) and U_Net, EDP model, ConvLSTM. The results show that the proposed model has better prediction performance. Through comparative model experiments, it is found that combining dilated convolution with the attention convolution module can not only improve the prediction performance of the model, but also achieve better results than using dilated convolution or the attention convolution module alone. And through the use of the attention convolution module, the parameters and running time added by dilated convolution to the ADC_Net model can be reduced to a certain extent, and the running efficiency can be improved.

## Supplementary Information

Supplementary Information.

## Data Availability

All data generated or analysed during this study are included in this published article [and its supplementary information files].
